# Neural correlates of individual differences in motor learning under reinforcement contexts

**DOI:** 10.1016/j.isci.2026.115336

**Published:** 2026-03-11

**Authors:** Hayato Otake, Naoki Senta, Junichi Ushiba, Mitsuaki Takemi

**Affiliations:** 1 Graduate School of Science and Technology, Keio University, Yokohama, Kanagawa 223-8522, Japan; 2 Faculty of Science and Technology, Keio University, Yokohama, Kanagawa 223-8522, Japan; 3 Graduate School of Advanced Science and Engineering, Hiroshima University, Higashi-Hiroshima, Hiroshima 739-8527, Japan

**Keywords:** Natural sciences, Biological sciences, Neuroscience, Clinical neuroscience

## Abstract

Rewards and punishments shape motor learning, yet individuals vary in their adaptation speed and skill retention. Previous studies have linked these processes to two electroencephalographic signatures: feedback-related negativity (FRN) and sensorimotor event-related desynchronization (ERD). However, their roles in individual learning differences remain unclear. We recorded electroencephalography while 64 adults performed a visuomotor rotation task where gains or losses scaled with movement error. Using Lasso regression, we examined whether these neural markers accounted for individual variability in learning and retention. Results demonstrated that the interaction between sensorimotor alpha-ERD during movement preparation in late adaptation and feedback condition explained retention. Stronger alpha-ERD predicted better retention only in the reward condition, whereas neither ERD nor FRN explained adaptation rates. These findings indicate that late-phase alpha-ERD reflects neural mechanisms supporting motor memory stabilization, which becomes behaviorally relevant specifically under positive reinforcement. Thus, pairing reward with interventions enhancing sensorimotor cortical excitability may facilitate skill maintenance.

## Highlights

• Sensorimotor alpha and beta ERD increased from early to late adaptation
• Feedback-related negativity decreased from early to late adaptation
• Late-phase alpha ERD was associated with skill retention in reward feedback condition
• Late-phase alpha-ERD may reflect neural mechanisms of motor memory stabilization

## Graphical abstract

**Figure:**
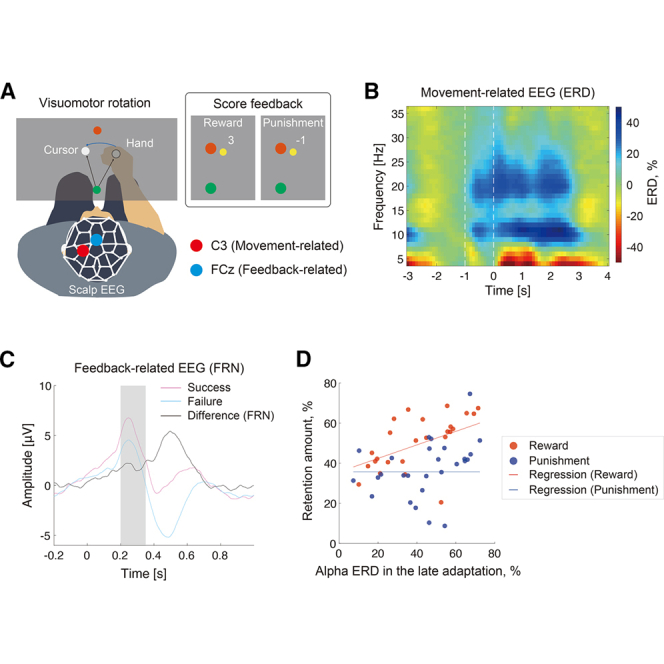

## Introduction

Rewards influence learning processes by shaping both procedural and declarative memory formation.^1^^,^^2^ They can be both positive and negative, and they activate the reward circuitry in the brain through the dopamine system.^3^ Comparing positive rewards and punishments (i.e., negative rewards), positive rewards increase the likelihood of repeating a behavior by reinforcing it and motivating continued effort, while punishments decrease the likelihood of a behavior by promoting avoidance of adverse outcomes.^4^ These differing motivational effects have been demonstrated in motor learning contexts, where rewards have been shown to reduce forgetting and enhance retention, whereas punishments can increase the speed of adaptation.^5^ However, these outcomes serve a dual role. They not only modulate motivation but also act as reinforcement feedback signals, conveying success or failure, that guide trial-by-trial learning.^6^

Importantly, individual variability is commonly observed in the extent to which rewards and punishments affect behavior, likely due to underlying neural differences. For example, inter-individual differences in reinforcement-based learning have been linked to baseline levels of striatal dopamine, with higher dopamine levels enhancing reward-driven learning and lower levels enhancing punishment-driven learning.^7^ Likewise, variability in neural responses to the same amount of monetary rewards has been reported during reinforcement learning tasks.^8^ Such variability in brain activity may also shape the extent of behavior modification in motor learning, including the speed of learning and retention of motor memory.^3^

Given the potential for rewards to enhance the effectiveness of interventions in motor rehabilitation and athletic training,^9^^,^^10^^,^^11^ it is important to understand the neural factors that drive individual differences in motor learning efficiency. To this end, we recorded scalp electroencephalogram (EEG) data while participants performed a visuomotor rotation task in which monetary rewards or punishments varied according to the size of their motor error.^5^ In this paradigm, monetary outcomes are inextricably linked to performance accuracy. Thus, while we manipulate the motivational context using rewards and punishments, we focus on the reinforcement learning signals (i.e., success and failure feedback) inherent in these outcomes to investigate the neural basis of learning. EEG offers millisecond temporal resolution for tracking neural dynamics during motor preparation, execution, and feedback phases. In this study, we focused on two EEG features with well-established functional interpretations. First, we examined alpha- and beta-band event-related desynchronization (ERD) recorded over the sensorimotor region (e.g., C3 electrode), which is commonly associated with engagement of human sensorimotor cortical excitability.^12^^,^^13^^,^^14^ Second, we analyzed the frontal feedback-related negativity (FRN), an event-related potential (ERP) linked to feedback processing.^15^^,^^16^^,^^17^ Together, these EEG features provide non-invasive proxies of sensorimotor engagement and feedback evaluation that can be related to individual differences in motor learning under reinforcement contexts.

In this study, we aimed to clarify the neural correlates of individual differences in motor learning under rewarding and punishing contexts. Specifically, we focused on how sensorimotor excitability and the processing of reinforcement feedback signals contribute to individual variability in motor adaptation and in the persistence of learned movements (retention). We quantified the learning amount as the degree of error compensation during adaptation and retention amount as the persistence of this compensation during the no-vision block. Based on prior work, we hypothesized that greater sensorimotor excitability (indexed by ERD) and enhanced processing of reinforcement signals (indexed by FRN in response to success/failure outcomes) would be associated with superior learning and retention performance. By testing these hypotheses, we sought to determine whether these neural processes independently account for inter-individual differences in motor learning. Consistent with the dual nature of monetary outcomes,^6^ we use the terms Reward and Punishment to denote the experimental manipulations, while specifically framing the neural analysis around the processing of reinforcement feedback signals.

## Results

**Figure 1:**
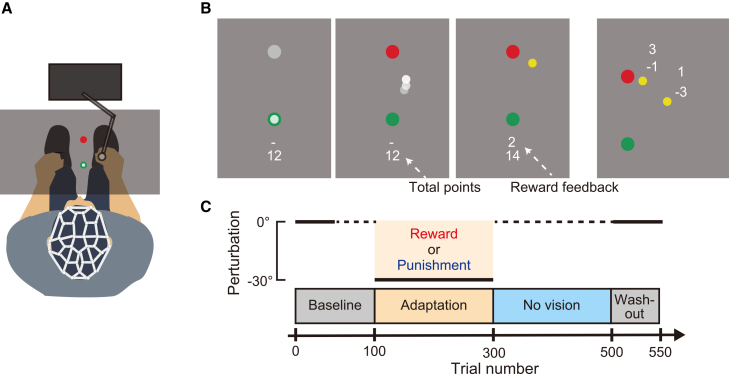
Experimental design (A) Experimental setup. Participants performed arm-reaching movements toward a visual target displayed on a monitor while wearing an EEG cap to record brain activity. (B) Experimental task. Reaching movements started from a green circle (starting position) to a red circle (target). During each trial, participants received online feedback of their hand position (white circle) as well as endpoint feedback (yellow circle). Reinforcement feedback (reward or punishment) was represented by positive or negative points based on endpoint error, with points accumulated throughout the adaptation block and displayed on the monitor. (C) Experimental paradigm. The task consisted of four blocks: baseline, adaptation, no vision, and washout. Online and endpoint feedback were provided for the baseline (first 50 trials), adaptation, and washout blocks. During the adaptation block, a 30° counterclockwise visuomotor rotation was introduced, and reward or punishment feedback was provided.

Sixty-four participants performed a visuomotor rotation task while their brain activity was recorded using scalp EEG (Figure 1A). Participants manipulated a cursor with a robotic manipulandum to make reaching movements toward a visual target displayed on a monitor (Figure 1B). The task consisted of four sequential blocks: baseline, adaptation, no vision, and washout (Figure 1C). During the adaptation block, a 30° counterclockwise visuomotor rotation was applied to assess adaptation to visuomotor perturbations. In the subsequent no-vision block, participants performed reaching movements without visual feedback, allowing us to evaluate the degree of motor memory retention.^5^

Participants were assigned to either a reward (*n* = 32) or punishment group (*n* = 32). In these groups, performance-dependent reinforcement feedback was provided in the form of positive points in the reward group and negative points in the punishment group (Table 1). These points accumulated throughout the adaptation block and were converted into monetary incentives. This design allows us to compare behavioral adaptation under different reinforcement contexts and to relate these behavioral outcomes to neural activity measured during task performance.

**Table 1:** Point system for reward and punishment conditions

Feedback condition	Hit target	<10° error	<20° error	<30° error	≥30° error
Reward	4 points	3 points	2 points	1 point	0 points
Punishment	0 points	−1 point	−2 points	−3 points	−4 points

Movements classified as “Fast” or “Slow” were considered equivalent to a ≥30° error. Such trials resulted in 0 points for the reward group and −4 points for the punishment group.

### Effects of reward and punishment on visuomotor rotation task performance

**Figure 2:**
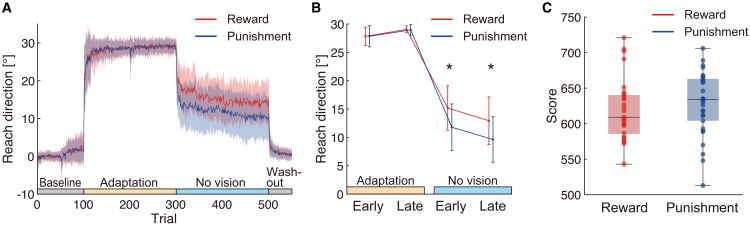
Effects of reward and punishment on motor adaptation and retention (A) Trial-by-trial angular reach direction during the visuomotor rotation task for the reward group (*n* = 29) and punishment group (*n* = 30). Solid lines indicate mean values. Shaded areas represent standard deviations. (B) Reach direction during the early and late phases of the adaptation and no-vision blocks. Significant differences between the reward and punishment groups were observed in both the early and late no-vision phases (∗*p* < 0.05). Data are represented as mean ± standard deviations. (C) Total scores for the reward and punishment groups. Boxplots display the median, interquartile range, and individual data points.

Figure 2A illustrates the results of the visuomotor rotation task. The adaptation and no-vision phases were further divided into early and late phases, and the mean angular reach directions were analyzed for these four phases (Figure 2B; see STAR Methods). A two-way mixed ANOVA with phase (early adaptation to late no vision) as the within-subject factor and feedback condition as the between-subject factor revealed a significant interaction effect between phase and feedback condition (reward vs. punishment). Mauchly’s test of sphericity indicated a violation of the sphericity assumption (*p* < 0.05). Consequently, the Greenhouse-Geisser correction was applied, confirming a significant interaction effect between phase and feedback condition (*F*(1.32, 75.06) = 7.31, *p* = 0.005). Post hoc pairwise comparisons identified significant differences between the reward and punishment groups in both early and late phases of the no-vision block (*p* < 0.05). Substantial inter-subject variability was observed in the no-vision phase, as reflected by the shaded areas in Figure 2A and the large error bars in Figure 2B. An independent sample *t* test revealed no significant difference in total scores between the reward and punishment groups (*t*(57) = 0.76, *p* = 0.45). Contrary to previous reports suggesting that punishment accelerates adaptation speed,^5^ our data showed that overall task performance during the adaptation phase was comparable between the two feedback conditions (Figure 2C).

### ERD changes during the adaptation task

During the adaptation block, scalp EEG signals were recorded continuously. ERD analyses were conducted on cleaned EEG data after preprocessing (see STAR Methods for details). Power spectral density was computed, and ERD was assessed relative to a baseline defined as the mean PSD during the −2 to −1 s period preceding the Go cue. ERD values were averaged over the left sensorimotor region (C3 and surrounding electrodes), with peak ERD identified during the preparatory phase of the movement (−1 to 0 s relative to the Go cue). These peak values were analyzed separately for the alpha (8–13 Hz) and beta (15–30 Hz) frequency bands and used for subsequent statistical analyses. EEG data from seven participants were excluded due to poor signal quality caused by movement artifacts or recording malfunctions, resulting in 57 participants being included in EEG analyses (see STAR Methods for details).

**Figure 3:**
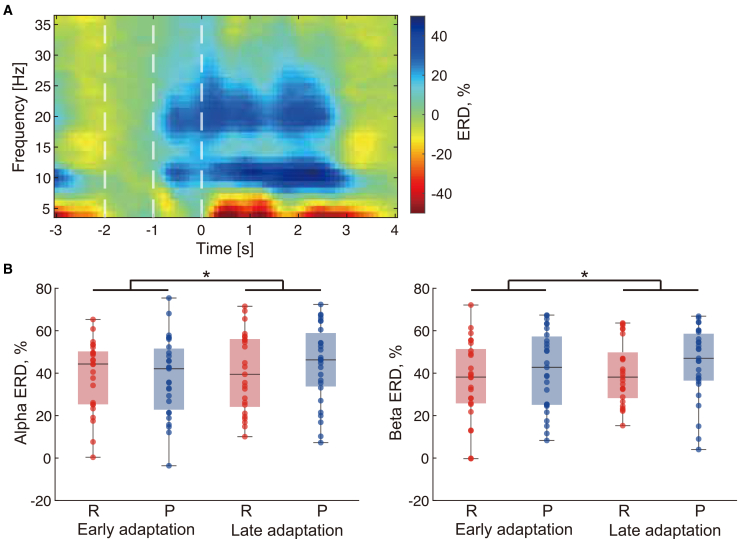
ERD during motor adaptation (A) Grand-average time-frequency map during the adaptation task, measured over the left sensorimotor region (C3). The preparatory phase (−1 to 0 s) is highlighted by white dotted lines. ERD was calculated relative to the baseline power spectrum (−2 to −1 s). (B) Alpha (8–13 Hz) and beta (15–30 Hz) ERD magnitudes during the preparatory phase in the early and late adaptation phases for both the reward group (R, *n* = 25) and punishment group (P, *n* = 27). Boxplots display the median, interquartile range, and individual data points. Significant main effects of time were found for both alpha and beta ERD (∗*p* < 0.05), suggesting a time-dependent change in ERD magnitude.

Figure 3A shows the time-frequency representation of ERD during the adaptation task, focusing on the alpha and beta frequency bands. ERD during the preparatory phase (−1 to 0 s relative to task onset) was observed across nearly all participants, irrespective of the reward or punishment condition (Figure 3B). Alpha and beta ERD during the early and late adaptation phases were compared between the reward and punishment groups. For alpha ERD, a two-way mixed ANOVA revealed a significant main effect of time (*F*(1, 50) = 4.90, *p* = 0.031), indicating a change in ERD magnitude between the early and late adaptation phases. However, there was no significant interaction between time and feedback condition (*F*(1, 50) = 2.02, *p* = 0.16), nor was there a significant main effect of feedback condition (*F*(1, 50) = 0.12, *p* = 0.73). Similarly, for beta ERD, a two-way mixed ANOVA revealed a significant main effect of time (*F*(1, 50) = 4.80, *p* = 0.033), suggesting a time-dependent change in ERD magnitude. However, no significant interaction between time and feedback condition was observed (*F*(1, 50) = 0.009, *p* = 0.92), and the main effect of feedback condition was also not significant (*F*(1, 50) = 0.97, *p* = 0.33). These results indicate that the observed ERD changes during the adaptation task were primarily driven by temporal factors rather than by the type of feedback condition.

### FRN changes during the adaptation task

To examine feedback-related neural responses during adaptation, ERPs were analyzed time-locked to the onset of the score feedback stimulus. Analyses focused on FRN measured over the fronto-central region.^17^^,^^18^^,^^19^ ERPs were derived from cleaned EEG data following preprocessing (see STAR Methods for details), and FRN waveforms were computed using a difference-wave approach contrasting successful and unsuccessful outcomes.^20^^,^^21^^,^^22^^,^^23^^,^^24^ FRN amplitude was quantified as the mean voltage within the 200–350 ms time window following feedback presentation and used for subsequent statistical analyses.^25^ FRN amplitudes exceeding ± 2SD from the group mean were treated as outliers and excluded (early: four participants; late: four participants; see STAR Methods).

**Figure 4:**
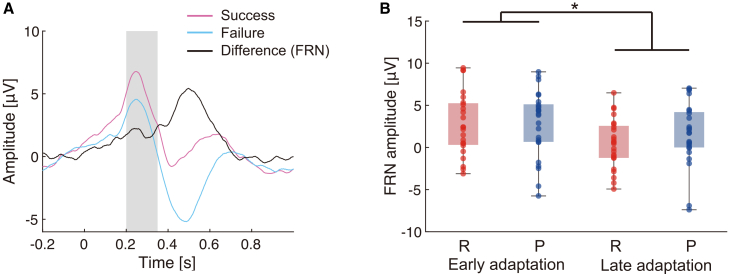
FRN during adaptation tasks (A) Grand-average waveforms of ERPs during the adaptation task. The black waveform represents FRN, calculated as the difference between ERPs for successful and unsuccessful trials. Time zero marks the onset of the reinforcement feedback presentation. The gray shaded area indicates the post-feedback window (200–350 ms) used for the FRN amplitude calculation. (B) FRN amplitudes during the early and late adaptation phases. Boxplots display the median, interquartile range, and individual data points of the reward group (R) and punishment group (P). A significant main effect of time was found (∗*p* < 0.05), suggesting a time-dependent reduction in FRN amplitudes. See also Figure S1 for the topographical distribution of FRN-related activity.

Figure 4A illustrates the grand-average ERPs and the corresponding FRN waveform (FCz). During the adaptation task, ERPs associated with FRN varied across participants. To confirm that the FRN response was spatially localized, we additionally inspected the topographical distribution of the FRN, which showed a fronto-central localization (Figure S1). A two-way mixed ANOVA revealed a significant main effect of time (*F*(1, 49) = 6.03, *p* < 0.05), indicating that FRN amplitudes changed between the early and late adaptation phases. However, no significant interaction was observed between time and feedback condition (*F*(1, 49) = 2.17, *p* = 0.15), and no significant main effect of feedback condition was detected (*F*(1, 49) = 0.078, *p* = 0.78). These findings suggest that changes in FRN amplitudes were influenced by task phase rather than the type of feedback condition.

### Neural correlates of learning and retention in motor adaptation

To identify neural correlates of individual differences in learning and retention, we first quantified behavioral learning and retention. Learning amount was defined as an index of motor memory acquisition based on error compensation during adaptation, whereas retention amount reflected the persistence of this compensation during the no-vision phase (see STAR Methods for definitions). We next conducted Lasso regression analyses. The following variables were included as regressors: feedback condition (categorical: reward = 1, punishment = 0), FRN amplitudes during early and late adaptation phases, alpha and beta ERD magnitudes during early and late adaptation, and the interaction terms between feedback condition and each EEG variable.

For the learning amount, the Lasso regression did not select any regressors, shrinking all coefficients to zero. This suggests that neither feedback processing responses (FRN) nor cortical excitability (ERD) reliably account for individual differences in the extent of error compensation in the current experimental design.

**Figure 5:**
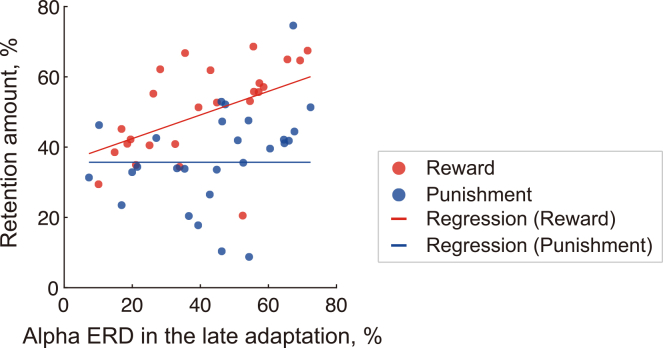
Neural correlates of retention in motor adaptation Scatterplot showing the relationship between alpha ERD in the late adaptation phase and retention amount, separated by feedback condition. Regression lines represent model fits for each group. Note that a significant association was observed specifically in the reward condition.

Regarding retention amount, Lasso regression identified a single non-zero coefficient corresponding to the interaction between alpha ERD magnitude during the late adaptation phase and the feedback condition. To assess statistical significance, this Lasso-selected interaction term was then entered into a final OLS model. The resulting model significantly explained the variance in retention amount (adjusted *R*^2^ = 0.31, *p* < 0.001; Figure 5):

$$Y=\beta_{0}+\beta_{1}X_{\text{interaction}}\;\left[\beta_{0}=35.7\;\left(p\;<\;0.001\right),\beta_{1}=0.34\;\left(p\;<\;0.001\right)\right]\text{,}$$

where ***Y*** represents the retention amount and ***X***_interaction_ is the interaction term (Late alpha ERD × Feedback condition). No significant main effects of either ERD or feedback condition were observed, suggesting that the relationship between late-phase sensorimotor cortex excitability and motor memory retention depends on the reinforcement context. Specifically, stronger alpha ERD magnitude was associated with better retention in the reward condition.

## Discussion

This study investigated the neural correlates of individual differences in motor learning under reinforcement contexts, with a focus on ERD over sensorimotor regions and FRN measured from frontal EEG channels. Lasso regression analyses revealed that the interaction between the feedback condition and alpha ERD magnitude in the late adaptation phase was a significant regressor of motor memory retention. This result implies that the relationship between alpha-band desynchronization and retention is not uniform across participants but instead depends on the reinforcement context (i.e., whether outcomes were framed as monetary gains or losses). Specifically, greater late alpha ERD magnitude was associated with better retention in the reward condition, whereas no corresponding association was observed in the punishment condition. These findings suggest that late alpha ERD functions as a condition-dependent neural correlate of retention, reflecting neural processes that are modulated by the reinforcement context.

### Alpha ERD in the late adaptation phase and its association with motor memory retention

Our results demonstrated that alpha ERD in the late adaptation phase was associated with motor memory retention specifically in the reward condition. This condition-dependent relationship suggests that the functional role of late-phase alpha ERD in memory stabilization may emerge clearly when successful outcomes carry positive reinforcement value. However, the precise functional role of alpha ERD in motor memory remains an open question. Below, we discuss several possible mechanisms that may explain why greater alpha ERD is linked to improved motor skill retention.

One possible explanation is that alpha ERD reflects increased sensorimotor cortical excitability,^12^^,^^13^ which may facilitate the retention of learned motor skills. This hypothesis aligns with previous findings, which showed that enhancing M1 excitability through anodal transcranial direct current stimulation during motor learning improves motor memory retention.^26^^,^^27^ In related work, Galea et al.^5^ reported that reward leads to greater retention than punishment and suggested that reward may enhance retention by providing dopaminergic reinforcement signals to M1, drawing on prior evidence that M1 contributes to the maintenance of adapted motor commands and receives projections from dopamine-producing neurons. Viewed in this context, the present finding—that the relationship between alpha ERD and retention emerged most clearly under the reward condition—may reflect the possibility that processing of positive reinforcement signals strengthen sensorimotor-dependent learning processes, thereby amplifying the behavioral relevance of sensorimotor excitability during learning.

Another potential mechanism involves the suppression of task-irrelevant information and the optimization of task-relevant neural activity. Alpha-band oscillations have been implicated in attentional filtering, selectively inhibiting distracting information while enhancing neural processing relevant to the task.^28^^,^^29^ This attentional modulation may contribute to the stabilization of motor memory, thereby improving skill retention. Importantly, reward has been shown to enhance attentional control and reduce interference from task-irrelevant information.^30^^,^^31^ Such reinforcement-driven increases in attentional engagement could amplify alpha-mediated filtering processes. From this perspective, the fact that the ERD-retention relationship emerged specifically in the reward condition may reflect that positive reinforcement heightened selective attention to successful movement outcomes, strengthening the formation and stabilization of sensorimotor representations.

Alternatively, stronger alpha ERD in the late adaptation phase may not cause, but rather reflect, improved motor memory retention. Individuals who develop more refined sensorimotor representations may exhibit greater ERD, which indicates increased activation of cortical neuronal networks,^32^ which in turn enhances motor memory retention. The significant increase in alpha and beta ERD from the early to the late adaptation phase observed in our study aligns with this interpretation. Prior research also indicates that sensorimotor cortical excitability, as indexed by ERD, increases with continued motor practice,^33^^,^^34^ possibly reflecting a shift from cognitively demanding control to more automatic execution. In the early adaptation phase, ERD tends to be modest as neural activity is primarily driven by error monitoring and rapid feedback-based adjustments.^16^ Over time, as learning progresses, motor control becomes more internally regulated, potentially giving rise to stronger ERD signals.^33^^,^^34^ This shift may reflect a consolidation process in which sensorimotor representations become more robust, facilitating the persistence of motor memory.

Although our findings established an association between alpha ERD and motor memory retention, the causal relationship remains unclear. An important next step is to determine whether direct modulation of alpha oscillations can enhance retention. This could be tested using non-invasive brain stimulation techniques such as rhythmic transcranial magnetic stimulation^35^ or transcranial alternating current stimulation,^36^ allowing for a causal assessment of alpha-band activity in motor memory consolidation.

### Dynamics of FRN during motor adaptation

The observed reduction in FRN amplitude over the adaptation phase suggests a gradual shift in learning strategies. As motor execution efficiency improves, participants may become less dependent on external reinforcement feedback.^16^ However, this reduction in FRN amplitude could also result from habituation rather than learning-related improvements. Given that FRN is most pronounced when errors are unexpected,^37^ a progressive decrease in error salience may contribute to its attenuation over time. An additional factor that may account for the reduced FRN amplitude is the change in feedback distribution across the task: participants tended to experience a higher frequency of successful outcomes in the later phase, reflecting improved performance (see Figure S2). Because FRN amplitude is sensitive to the relative proportion of positive and negative outcomes,^38^ the observed decline in FRN amplitude may therefore reflect this change in outcome frequency. Distinguishing between these possibilities remains a challenge and warrants further investigation.

Although the FRN component exhibited a frontocentral distribution consistent with prior literature, it did not emerge as a reliable regressor for individual differences in learning outcomes in the present study. One possibility is that FRN amplitude was strongly influenced by the change in outcome frequency across the task,^38^ which may have masked trait-level variability relevant to learning. Another possibility is that, although an FRN was observable at the group level, its trial-level consistency within individuals may not have been sufficient for it to serve as a stable regressor of learning performance. In particular, variability in signal-to-noise ratio across participants or phases could have reduced the reliability of the FRN measure, as ERP components must be clearly distinguishable from background EEG activity to function as robust individual-difference markers.^39^ More broadly, FRN reflects rapid, surprise-driven evaluative signals, which may make it less suited than ERD for capturing stable individual differences in motor adaptation. Future work will be needed to clarify under which task conditions FRN more robustly relates to learning performance.

### Conclusions

This study demonstrates that individual differences in motor learning under reinforcement contexts are shaped not only by behavioral performance but also by intrinsic neural dynamics expressed during the late phase of adaptation. In particular, the interaction between the reinforcement context and alpha-band ERD during movement preparation emerged as a significant regressor of motor retention, suggesting that positive reinforcement signals engage sensorimotor processes that help stabilize acquired motor commands. More broadly, our findings underscore the value of EEG markers for understanding why individuals differ in their ability to acquire and retain motor skills. By demonstrating that late-phase neural activity is associated with retention specifically in the reward condition, this study provides a framework for refining reinforcement-based training strategies. These results suggest that optimizing motor learning and retention may involve integrating reward-based training with interventions that enhance sensorimotor cortical excitability, such as non-invasive brain stimulation. This approach holds promises for applications in rehabilitation and athletic training.

### Limitations of the study

A key limitation of this study is the absence of punishment-induced acceleration in learning, which has been reported in previous research.^5^ One possible explanation is that, although participants received negative score feedback, they may have relied more on sensory error feedback, which would reduce the influence of punishment. This suggests that punishment feedback may not have been a primary driver of learning for most individuals; however, because this interpretation is speculative, it warrants direct testing in future work. Additionally, how punishment was framed may have affected its effectiveness. Although both our study and previous research^5^ employed a deduction from an initial endowment strategy, the feedback presentation differed. Unlike previous studies where losses were displayed directly in monetary currency, our study used a points-based system. This indirect framing may have been perceived as less salient or consequential than direct monetary loss.

Furthermore, we must acknowledge a limitation regarding the conceptual framing of our experimental design. As pointed out in recent work,^6^ standard reward-based motor learning tasks, including ours, often conflate the motivational effects of monetary reward with the informational value of performance feedback (i.e., reinforcement signals). In our design, monetary “Reward” trials always coincided with “Success” feedback, and “Punishment” trials coincided with “Failure” feedback. Consequently, we cannot strictly disentangle whether the observed neural modulations were driven by the monetary incentive itself, the positive reinforcement signal, or a combination of both. Future studies using designs that orthogonally manipulate outcome valence and monetary value are needed to isolate these factors.

Another limitation is the spatial resolution of EEG, which restricts its ability to assess activity in deep brain structures such as the striatum, a key region in reward-based learning. While EEG provides valuable insights into cortical activity, it cannot directly capture neural processes within subcortical structures that may contribute to individual differences in learning and retention. Future studies could use functional MRI or transcranial focused ultrasound stimulation to complement EEG findings and investigate how reward processing in deeper brain regions influences motor learning outcomes.^40^

A further limitation concerns the sex imbalance in our sample. Some studies have reported sex-related differences in ERP^41^^,^^42^ and ERD.^43^^,^^44^ Because our study was not designed to examine sex as a factor, we cannot exclude the possibility that sex-related variability influenced the neural-behavioral relationships observed here. However, given that our main results were driven largely by within-subject changes across learning phases and by continuous neural measures (ERD and FRN), any systematic bias introduced by gender differences is likely minimized. Future work with more balanced samples will be important for evaluating the generalizability of these findings.

## Resource availability

### Lead contact

Requests for further information and resources should be directed to the lead contact, Mitsuaki Takemi (mitsuaki1988@me.com).

### Materials availability

Not applicable.

### Data and code availability

• Behavioral data and subject-level derived EEG data required to reproduce all figures and statistical analyses reported in this study are publicly available in the OSF repository (https://doi.org/10.17605/OSF.IO/4JBU5). Raw EEG data cannot be made publicly available due to restrictions imposed by the original ethics approval and informed consent obtained from participants. Anonymized raw EEG data are available from the lead author upon reasonable request.
• All analysis codes used in this study are publicly available in the OSF repository (https://doi.org/10.17605/OSF.IO/4JBU5).
• No additional resources were generated in this study.

## Acknowledgments

The authors thank Dr. Seitaro Iwama (Faculty of Science and Technology, Keio University) for his valuable comments on this work. This work was supported by JST Moonshot R&D (#JPMJMS2012) to J.U. and M.T.

## Author contributions

H.O., formal analysis, investigation, software, visualization, and writing – original draft; N.S., methodology, software, validation, visualization, and writing – review and editing; J.U., funding acquisition, resources, supervision, and writing – review and editing; M.T., conceptualization, funding acquisition, project administration, supervision, and writing – review and editing.

## Declaration of interests

J.U. is a founder and CEO of the University Startup Company, LIFESCAPES Inc., which focuses on the research, development, and sales of rehabilitation devices, including brain-computer interface. He receives a salary from LIFESCAPES Inc. and holds shares in the company. This company does not have any relationship with the device or setup used in the present study.

## Declaration of generative AI and AI-assisted technologies in the writing process

During the preparation of this work, the authors used ChatGPT (OpenAI) to assist with the translation of text from the authors’ native language into English, manuscript editing (including grammar and vocabulary), and partial implementation of data analysis code. After using this tool, the authors reviewed and edited the content as needed, and they take full responsibility for the content of the publication.

## STAR★Methods

### Key resources table

**Table:** 

REAGENT or RESOURCE	SOURCE	IDENTIFIER
**Deposited data**		

Behavioral data	This paper	https://doi.org/10.17605/OSF.IO/4JBU5
Subject-level derived EEG data	This paper	https://doi.org/10.17605/OSF.IO/4JBU5

**Software and algorithms**		

Analysis code	This paper	https://doi.org/10.17605/OSF.IO/4JBU5
G∗Power 3.1	Faul et al.^45^	https://www.psychologie.hhu.de/arbeitsgruppen/allgemeine-psychologie-und-arbeitspsychologie/gpower
MATLAB R2023a	MathWorks	https://www.mathworks.com/products/matlab.html
FieldTrip toolbox	Oostenveld et al.^46^	https://www.ru.nl/neuroimaging/fieldtrip/
JASP version 0.18.3	JASP Team	https://jasp-stats.org/

### Experimental model and study participant details

Sixty-four young adults (24 ± 5 years, 12 females) participated in this study. All participants were right-handed, had no history of neurological or psychiatric diseases, and were not on chronic medication. Five participants who responded on the questionnaire that they were unable to perform the task properly due to drowsiness or lack of motivation were excluded from the results. Data from seven participants were excluded from the EEG analysis due to poor signal quality caused by body movements or other noise (n = 4) or malfunctions in the EEG recording system (n = 3), although their data were included in the behavioral analysis. The study was conducted in accordance with the Declaration of Helsinki, and the research protocol was approved by the ethics committee of the Faculty of Science and Technology at Keio University (IRB approval numbers: 2023-071 and 2024-010). All participants provided written informed consent.

### Method details

#### Experimental apparatus and setup

Scalp EEG signals were recorded using an EEG System (GES 400; Electrical Geodesics, Inc., Oregon, USA) equipped with a 128-channel HydroCel Geodesic Sensor Net (HCGSN-128) at a sampling rate of 1,000 Hz. The ground and reference electrodes were positioned at CPz and Cz, respectively, following the extended 10-20 system. Electrode impedance was maintained below 50 kΩ. The EEG signals were filtered online using a band-pass filter (0.1 to 70 Hz) and a notch filter (50 Hz).

A custom-designed program developed in C++ was used to implement the motor task. Participants sat with their chin resting on a chin rest and grasped the handle of a robotic manipulandum (Phantom Premium 1.5HF, SensAble Technologies, Wilmington, MA) with their right hand in a semi-pronated forearm posture (Figure 1A). A computer monitor (1920 × 1080 pixels) positioned above the manipulandum appeared to be in the same plane as the participant’s hand, which effectively obscured direct visual feedback of the hand position. Instead, a cursor representing the hand’s position was displayed on the monitor, with its positional information calculated based on the manipulandum’s movements. Handle position data were recorded at a sampling rate of 1,000 Hz.

#### Motor task and experimental procedure

The motor task required participants to manipulate a cursor displayed on the monitor using a robotic manipulandum to perform shooting movements toward a visual target (Figure 1B). At the beginning of each trial, participants moved a white cursor (0.3 cm diameter) into a green circle (1 cm diameter) representing the starting position. Once the cursor was held within the starting position for more than 1.5 s, the monitor displayed a gray target (0.5 cm diameter) located 8 cm in front of the starting position. After a fixed interval of 1 s, the target changed color from gray to red, serving as the Go cue. Participants were instructed to make a fast and accurate shooting movement through the target to minimize online corrections during the movement. When the movement stopped, the location where the cursor crossed the invisible boundary of an 8-cm radius circle centered on the starting position was displayed with a yellow circle to indicate the endpoint error. Additionally, feedback regarding movement speed was provided. Movements with maximum speeds below 312 mm/s were labeled as “Slow,” while those exceeding 469 mm/s were labeled as “Fast.” After each trial, participants moved the cursor back to the starting position.^5^

The experiment consisted of four blocks: baseline, adaptation, no vision, and washout (Figure 1C), with visual feedback varying across blocks. During the baseline block (trials 1 to 100), the cursor was visible, and endpoint feedback was provided for the first 50 trials; however, both cursor and endpoint feedback were removed in the latter 50 trials. In the adaptation block (trials 101 to 300), a 30° counterclockwise visuomotor rotation was applied, causing the cursor’s movement to deviate from the hand’s movement. This visuomotor transformation introduced a motor error, which required participants to modify their hand trajectories to compensate for the altered environment. In the no-vision block (trials 301 to 500), participants performed reaching movements without any visual feedback. During this phase, no information about movement accuracy was provided, allowing hand trajectories to gradually return to baseline, thereby characterizing the degree of memory retention. Finally, the washout block (trials 501 to 550) replicated the conditions of the first 50 trials of the baseline block.

To prevent fatigue, participants were given short rest periods of less than 3 min every 100 trials. During these breaks, they were instructed to keep their arms beneath the monitor. EEG signals were recorded continuously throughout all experimental blocks.

#### Visuomotor rotation with reward and punishment feedback

Participants were divided into two groups: the reward group (*n* = 32) and the punishment group (*n* = 32). Participants were assigned to each condition according to an allocation schedule generated prior to data collection. The allocation sequence was created by MT, and HO enrolled participants and conducted the experiments in accordance with this schedule. During the adaptation block, the type of feedback varied depending on the group, with the reward group receiving positive feedback and the punishment group receiving negative feedback (Table 1). The reward group started with 0 points, while the punishment group started with 800 points. Both groups could earn a maximum of 800 points and a minimum of 0 points, with points accumulating throughout the block. The reward group accumulated positive points, while the punishment group accumulated negative points. Participants in both groups were shown the points they earned on a trial-by-trial basis, along with the total accumulated points for the block. The total points accumulated by the end of the block were converted into Japanese yen at a rate of three yen per point, and the resulting amount was added to their participation wage. All participants were informed that closer reaches to the target would result in a greater monetary incentive, and their final score would determine their additional compensation. They were also made aware of the maximum and minimum points they could earn, as well as the conversion rate of points to yen. Participants received a base wage of 1,000 yen/h, to which their bonus earnings were added.

#### Behavioral data analysis

Behavioral data analysis was conducted using MATLAB R2023a (MathWorks, Natick, MA, USA). For each trial, the angular reach direction was calculated as the angular difference between hand and target positions at the point where the cursor crossed the 8-cm invisible circle centered on the starting position. Under veridical feedback conditions, the ideal reach direction was 0°. However, during the visuomotor transformation phase, participants were required to compensate for the rotation. For example, with a -30° counterclockwise visuomotor rotation, accurate compensation required a 30° clockwise reach direction.

To quantify motor learning and retention, we defined two metrics: the learning amount, which reflects the degree of motor memory acquisition, and the retention amount, which reflects the degree of motor memory retention. These metrics were calculated using the following equations:

$$Learning\;amount=\frac{\theta \left(n_{LA}\right)}{30}\times 100,\%$$

$$Retention\;amount=\frac{\theta \left(n_{NV}\right)}{30}\times 100,\%$$

Here, *θ*(*n*) represents the reaching direction at trial *n*, *n*_*LA*_ refers to trials in the late adaptation phase (trials 201 to 300), and *n*_*NV*_ refers to trials in the no-vision phase (trials 301 to 500).

#### EEG preprocessing

EEG analysis was performed using MATLAB R2023a and the FieldTrip toolbox.^46^ Raw EEG signals were filtered offline using a 4th-order Butterworth filter, with different frequency ranges applied depending on the analysis: 0.1 to 30 Hz for FRN analysis and 1 to 60 Hz for ERD analysis. Noisy channels were excluded based on their power spectral density and amplitude range (15.2 ± 6.2 channels for ERD and 13.0 ± 6.1 for FRN, out of 128 total channels). A common average reference was first applied as a spatial filter to reduce noise and improve signal quality. Independent component analysis was then conducted to remove artifacts caused by eye movements and muscle activity. Artifact-related independent components were manually identified and subtracted from the EEG signals. Spatial interpolation was conducted using the FieldTrip toolbox “ft_channelrepair” function to reconstruct signals for the excluded channels. This function uses weighted data from neighboring channels. For the FRN analysis, the preprocessed data were subsequently re-referenced to the average of the left and right mastoids to enhance the visibility of frontocentral ERP components. ERD analysis was conducted using the common average reference.

#### Event-related desynchronization analysis

Cleaned EEG data were segmented into 7 s epochs, time-locked to the Go cue (-3 to +4 s). Trials containing artifacts were manually identified by visual inspection and excluded (15 ± 6.2% per participant). The power spectral density (PSD) was calculated using the FieldTrip toolbox “ft_freqanalysis” function with the multitaper convolution (*mtmconvol*) method. For PSD computation, a 1 s Hanning-tapered sliding window was used, advancing in 0.1-s steps (90% overlap) to provide high temporal resolution for ERD analysis. ERD was computed using the following equation:

$$ERD\left(f,t\right)=\frac{R\left(f\right)-A\left(f,t\right)}{R\left(f\right)}\times 100$$

Here, *R*(*f*) represents the average PSD during the reference period (-2 to -1 s relative to the Go cue) at a frequency *f*, and A(*t*, *f*) denotes the PSD at a given time point *t*.

ERD values were calculated for each participant by averaging data over the left sensorimotor region (C3) and its six surrounding channels. The maximum ERD values were identified within the time range of -1 to 0 s relative to the Go cue, corresponding to the preparatory phase of the reaching movement. These maximum ERD values were analyzed separately for the alpha band (8 to 13 Hz) and beta band (15 to 30 Hz). Average ERD values were then computed within a ±0.2 s time window and ±2 Hz frequency range centered on the maximum ERD values to account for inter-individual variability in the temporal and spectral characteristics of the peak ERD. The resulting averaged ERD values were subsequently used for statistical analyses.

#### Feedback-related negativity analysis

FRN is most prominently observed in EEG signals recorded from FCz^17^^,^^18^^,^^19^ and is commonly analyzed using the difference-wave approach in ERPs. FRN is classically characterized by a negative voltage deflection following non-reward feedback. However, recent studies suggest that reward-related positive responses, while not forming a distinct peak alone, also contribute to FRN when analyzed using the difference-wave approach. This analysis allows for a more accurate representation.^20^^,^^21^^,^^22^^,^^23^^,^^24^

In this study, cleaned EEG signals recorded from FCz and its surrounding 7 electrodes were segmented into 0.8 s epochs, time-locked to the onset of the score feedback stimulus (-200 to +600 ms). Trials containing artifacts were manually identified and excluded (1.6 ± 3.8% per participant). ERPs were calculated by separately averaging the EEG time series across trials for successful outcomes (reward group: 4 points; punishment group: 0 points) and unsuccessful outcomes (reward group: 3 to 0 points; punishment group: -1 to -4 points). Baseline correction was applied to all ERPs by subtracting the average voltage during the 200 ms period immediately preceding the onset of the score feedback from each trial. After applying baseline correction to individual trials, ERPs were averaged across all trials within each condition (e.g., successful and unsuccessful outcomes) to calculate condition-specific waveforms. For each participant, difference waves were calculated by subtracting ERPs for unsuccessful outcomes from those for successful outcomes. The FRN amplitude was then determined as the mean voltage of the difference waves within the 200 to 350 ms time window following the presentation of score feedback, in line with a previously established protocol.^25^ Finally, FRN amplitudes that fell beyond ± 2SD of the group mean were treated as outliers and excluded from further analysis (early: 4 participants; late: 4 participants).

### Quantification and statistical analysis

#### Sample size estimation and power analysis

The target sample size was determined *a priori* using G∗Power 3.1.^45^ Effect-size estimates were derived from two relevant EEG studies. Palidis et al.^25^ reported *R*^2^ = 0.242 for a multiple-regression model linking FRN components to motor learning performance, whereas Pollok et al.^47^ found a correlation of *r* = -0.67 (*r*^2^ ≈ 0.45) between beta band ERD changes and reaction-time improvement. We assumed that there were modest inter-regressor correlations among the seven planned regressors (feedback condition plus six EEG variables related to FRN and alpha/beta ERD across early and late adaptation) and conservatively set the expected overall coefficient of determination to *R*^2^ = 0.25 (equivalent to *f*^2^ = 0.33). A fixed-model multiple-regression power analysis (*α* = 0.05, 1-*β* = 0.80, 7 regressors) indicated a required sample of 51 participants. We anticipated a 20% data loss based on a pilot experiment (5% attrition and 15% unusable EEG recordings), so we recruited 64 participants to preserve the desired statistical power.

#### Statistical analysis of behavioral and EEG data

Statistical analyses were conducted using JASP (version 0.18.3; https://jasp-stats.org/). To compare reach direction, ERD values, and FRN amplitudes between reward and punishment feedback conditions and across experimental blocks, a two-way ANOVA was conducted with block as the within-subject factor and feedback condition as the between-subject factor. Prior to conducting ANOVA, we assessed the sphericity assumption using Mauchly’s test. If the sphericity assumption was violated, we applied the Greenhouse–Geisser correction to adjust for the degrees of freedom. If ANOVA yielded statistically significant main effects or feedback × block interactions, post-hoc t-tests were performed with Bonferroni–Holm correction to compare specific condition or block differences. A p-value of less than 0.05 was considered statistically significant. Detailed statistical information related to all figures is provided in Data S1.

#### Regression analysis of neural activity and individual differences

To examine the relationship between neural activity and individual differences in learning and retention, we employed Lasso regression implemented in MATLAB R2023a. Candidate regressors included the feedback condition (categorical), EEG features (FRN, alpha/beta ERD in early/late phases), and the interaction terms between feedback condition and each EEG feature. The regularization parameter (lambda) was determined via 5-fold cross-validation using the 1SE rule. Variables with non-zero coefficients were then used to construct a final ordinary least squares (OLS) model to assess statistical significance.

## Supplemental information

Document S1. Figures S1 and S2

Data S1. Statistical information relative to the figures

## References

[1] Chen X., Holland P., Galea J.M. (2018). The effects of reward and punishment on motor skill learning. Curr. Opin. Behav. Sci..

[2] Miendlarzewska E.A., Bavelier D., Schwartz S. (2016). Influence of reward motivation on human declarative memory. Neurosci. Biobehav. Rev..

[3] Zhao J., Guo J., Chen Y., Li W., Zhou P., Zhu G., Han P., Xu D. (2024). Improving rehabilitation motivation and motor learning ability of stroke patients using different reward strategies: study protocol for a single-center, randomized controlled trial. Front. Neurol..

[4] Schultz W., Dayan P., Montague P.R. (1997). A neural substrate of prediction and reward. Science.

[5] Galea J.M., Mallia E., Rothwell J., Diedrichsen J. (2015). The dissociable effects of punishment and reward on motor learning. Nat. Neurosci..

[6] Vassiliadis P., Derosiere G., Dubuc C., Lete A., Crevecoeur F., Hummel F.C., Duque J. (2021). Reward boosts reinforcement-based motor learning. iScience.

[7] Cools R., Frank M.J., Gibbs S.E., Miyakawa A., Jagust W., D'Esposito M. (2009). Striatal dopamine predicts outcome-specific reversal learning and its sensitivity to dopaminergic drug administration. J. Neurosci..

[8] Kim S.H., Yoon H., Kim H., Hamann S. (2015). Individual differences in sensitivity to reward and punishment and neural activity during reward and avoidance learning. Soc. Cogn. Affect. Neurosci..

[9] Grau-Sánchez J., Münte T.F., Altenmüller E., Duarte E., Rodríguez-Fornells A., Rubio F., Altenmüller E., Rodríguez-Fornells A. (2020). Potential benefits of music playing in stroke upper limb motor rehabilitation. Neurosci. Biobehav. Rev..

[10] Quattrocchi G., Greenwood R., Rothwell J.C., Galea J.M., Bestmann S. (2017). Reward and punishment enhance motor adaptation in stroke. J. Neurol. Neurosurg. Psychiatry.

[11] Zhao J., Zhang G., Xu D. (2024). The effect of reward on motor learning: different stage, different effect. Front. Hum. Neurosci..

[12] Pfurtscheller G., Lopes da Silva F.H. (1999). Event-related EEG/MEG synchronization and desynchronization: basic principles. Clin. Neurophysiol..

[13] Takemi M., Masakado Y., Liu M., Ushiba J. (2013). Event-related desynchronization reflects downregulation of intracortical inhibition in human primary motor cortex. J. Neurophysiol..

[14] Takemi M., Maeda T., Masakado Y., Siebner H.R., Ushiba J. (2018). Muscle-selective disinhibition of corticomotor representations using a motor imagery-based brain-computer interface. Neuroimage.

[15] Gehring W.J., Willoughby A.R. (2002). The medial frontal cortex and the rapid processing of monetary gains and losses. Science.

[16] Holroyd C.B., Coles M.G.H. (2002). The neural basis of human error processing: Reinforcement learning, dopamine, and the error-related negativity. Psychol. Rev..

[17] Miltner W.H., Braun C.H., Coles M.G. (1997). Event-related brain potentials following incorrect feedback in a time-estimation task: evidence for a “generic” neural system for error detection. J. Cogn. Neurosci..

[18] Holroyd C.B., Krigolson O.E. (2007). Reward prediction error signals associated with a modified time estimation task. Psychophysiology.

[19] Pfabigan D.M., Alexopoulos J., Bauer H., Sailer U. (2011). Manipulation of feedback expectancy and valence induces negative and positive reward prediction error signals manifest in event-related brain potentials. Psychophysiology.

[20] Baker T.E., Holroyd C.B. (2011). Dissociated roles of the anterior cingulate cortex in reward and conflict processing as revealed by the feedback error-related negativity and N200. Biol. Psychol..

[21] Becker M.P.I., Nitsch A.M., Miltner W.H.R., Straube T. (2014). A single-trial estimation of the feedback-related negativity and its relation to BOLD responses in a time-estimation task. J. Neurosci..

[22] Carlson J.M., Foti D., Mujica-Parodi L.R., Harmon-Jones E., Hajcak G. (2011). Ventral striatal and medial prefrontal BOLD activation is correlated with reward-related electrocortical activity: A combined ERP and fMRI study. Neuroimage.

[23] Heydari S., Holroyd C.B. (2016). Reward positivity: Reward prediction error or salience prediction error?. Psychophysiology.

[24] Walsh M.M., Anderson J.R. (2012). Learning from experience: Event-related potential correlates of reward processing, neural adaptation, and behavioral choice. Neurosci. Biobehav. Rev..

[25] Palidis D.J., Cashaback J.G.A., Gribble P.L. (2019). Neural signatures of reward and sensory error feedback processing in motor learning. J. Neurophysiol..

[26] Galea J.M., Vazquez A., Pasricha N., de Xivry J.J.O., Celnik P. (2011). Dissociating the roles of the cerebellum and motor cortex during adaptive learning: the motor cortex retains what the cerebellum learns. Cereb. Cortex.

[27] Reis J., Schambra H.M., Cohen L.G., Buch E.R., Fritsch B., Zarahn E., Celnik P.A., Krakauer J.W. (2009). Noninvasive cortical stimulation enhances motor skill acquisition over multiple days through an effect on consolidation. Proc. Natl. Acad. Sci. USA.

[28] Klimesch W., Sauseng P., Hanslmayr S. (2007). EEG alpha oscillations: The inhibition–timing hypothesis. Brain Res. Rev..

[29] Jensen O., Mazaheri A. (2010). Shaping functional architecture by oscillatory alpha activity: Gating by inhibition. Front. Hum. Neurosci..

[30] Krebs R.M., Boehler C.N., Woldorff M.G. (2010). The influence of reward associations on conflict processing in the Stroop task. Cognition.

[31] Padmala S., Pessoa L. (2011). Reward reduces conflict by enhancing attentional control and biasing visual cortical processing. J. Cogn. Neurosci..

[32] Neuper C., Pfurtscheller G. (2001). Event-related dynamics of cortical rhythms: Frequency-specific features and functional correlates. Int. J. Psychophysiol..

[33] Gehringer J.E., Arpin D.J., Heinrichs-Graham E., Wilson T.W., Kurz M.J. (2019). Practice modulates motor-related beta oscillations differently in adolescents and adults. J. Physiol..

[34] Nelson A.B., Moisello C., Lin J., Panday P., Ricci S., Canessa A., Di Rocco A., Quartarone A., Frazzitta G., Isaias I.U. (2017). Beta Oscillatory Changes and Retention of Motor Skills during Practice in Healthy Subjects and in Patients with Parkinson’s Disease. Front. Hum. Neurosci..

[35] Thut G., Veniero D., Romei V., Miniussi C., Schyns P., Gross J. (2011). Rhythmic TMS causes local entrainment of natural oscillatory signatures. Curr. Biol..

[36] Wach C., Krause V., Moliadze V., Paulus W., Schnitzler A., Pollok B. (2013). Effects of 10 Hz and 20 Hz transcranial alternating current stimulation (tACS) on motor functions and motor cortical excitability. Behav. Brain Res..

[37] Heldmann M., Rüsseler J., Münte T.F. (2008). Internal and external information in error processing. BMC Neurosci..

[38] Krigolson O.E. (2018). Event-related brain potentials and the study of reward processing: Methodological considerations. Int. J. Psychophysiol..

[39] Picton T.W., Bentin S., Berg P., Donchin E., Hillyard S.A., Johnson R., Miller G.A., Ritter W., Ruchkin D.S., Rugg M.D., Taylor M.J. (2000). Guidelines for using human event-related potentials to study cognition: Recording standards and publication criteria. Psychophysiology.

[40] Nakajima K., Osada T., Ogawa A., Tanaka M., Oka S., Kamagata K., Aoki S., Oshima Y., Tanaka S., Konishi S. (2022). A causal role of anterior prefrontal-putamen circuit for response inhibition revealed by transcranial ultrasound stimulation in humans. Cell Rep..

[41] Gölgeli A., Süer C., Ozesmi C., Dolu N., Aşcioğlu M., Sahin O. (1999). The effect of sex differences on event-related potentials in young adults. Int. J. Neurosci..

[42] Riel H., Lee J.B., Fisher D.J., Tibbo P.G. (2019). Sex differences in event-related potential (ERP) waveforms of primary psychotic disorders: A systematic review. Int. J. Psychophysiol..

[43] Fujimoto T., Okumura E., Kodabashi A., Takeuchi K., Otsubo T., Nakamura K., Yatsushiro K., Sekine M., Kamiya S., Shimooki S., Tamura T. (2016). Sex Differences in Gamma Band Functional Connectivity Between the Frontal Lobe and Cortical Areas During an Auditory Oddball Task, as Revealed by Imaginary Coherence Assessment. Open Neuroimag. J..

[44] Korzhyk O., Morenko O., Morenko A., Kotsan I. (2019). Gender differences in brain processes during inhibition of manual movements programs. Ann. Neurosci..

[45] Faul F., Erdfelder E., Buchner A., Lang A.-G. (2009). Statistical power analyses using G∗Power 3.1: Tests for correlation and regression analyses. Behav. Res. Methods.

[46] Oostenveld R., Fries P., Maris E., Schoffelen J.-M. (2011). FieldTrip: Open source software for advanced analysis of MEG, EEG, and invasive electrophysiological data. Comput. Intell. Neurosci..

[47] Pollok B., Latz D., Krause V., Butz M., Schnitzler A. (2014). Changes of motor-cortical oscillations associated with motor learning. Neuroscience.

