# Supplementary material for: Neural correlates of individual differences in motor learning under reinforcement contexts
Source: iScience. 2026 Mar 11;29(4):115336. doi: 10.1016/j.isci.2026.115336 (PMC13049676; doi:10.1016/j.isci.2026.115336)
Supplement: Document S1. Figures S1 and S2 [file mmc1.pdf]

iScience, Volume *29*

Supplemental information

Neural correlates of individual differences in motor learning under reinforcement contexts

Hayato Otake, Naoki Senta, Junichi Ushiba, and Mitsuaki Takemi

Supplementary materials

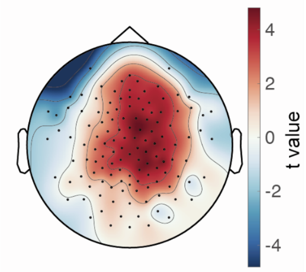

Supplementary Figure 1. Scalp topography of FRN-related activity, related to Figure 4. Scalp topography is shown as t-values contrasting successful and unsuccessful outcomes during the adaptation blocks.

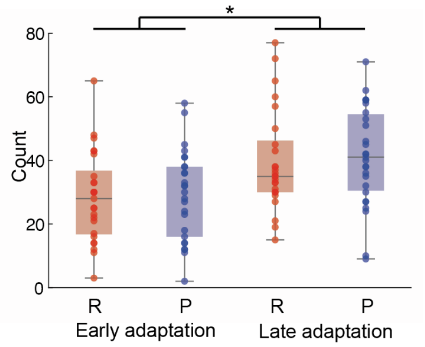

Supplementary Figure 2. Number of successful trials across adaptation phases, related to Discussion. The number of successful trials during the early and late adaptation phases for the reward group (R) and punishment group (P). Box plots display the median, interquartile range, and individual data points. A significant main effect of time was observed, indicating that participants experienced a higher frequency of successful trials in the late phase than in the early phase.
